# Entanglement and the density matrix renormalization group in the generalized Landau paradigm

**DOI:** 10.1038/s41567-025-02961-2

**Published:** 2025-08-15

**Authors:** Laurens Lootens, Clement Delcamp, Frank Verstraete

**Affiliations:** 1 Department of Applied Mathematics and Theoretical Physics, University of Cambridge, Cambridge, UK; 2 Department of Physics and Astronomy, Ghent University, Ghent, Belgium; 3 Institut des Hautes Études Scientifiques, Bures-sur-Yvette, France

**Keywords:** Quantum information, Theoretical physics

## Abstract

The fields of entanglement theory and tensor networks have recently emerged as central tools for characterizing quantum phases of matter. Here we determine the entanglement structure of ground states of gapped symmetric quantum lattice models and use this to obtain the most efficient tensor network representation of those ground states. We do this by showing that degeneracies in the entanglement spectrum arise through a duality transformation of the original model to the unique dual model where the entire dual symmetry is spontaneously broken. Physically, this duality transformation amounts to a—potentially twisted—gauging of the unbroken symmetry in the original ground state. In general, the dual symmetries of the resulting models are generalized non-invertible symmetries that cannot be described by groups. This result has strong implications for the complexity of simulating many-body systems using variational tensor network methods. For every phase in the phase diagram, the dual representation of the ground state that completely breaks the symmetry minimizes both the entanglement entropy and the required number of variational parameters. We demonstrate the applicability of this idea by developing a generalized density matrix renormalization group algorithm that works on constrained Hilbert spaces and quantify the computational gains obtained over traditional tensor network methods in a perturbed Heisenberg model. Our work testifies to the usefulness of generalized non-invertible symmetries and their formal category theoretic description for the practical simulation of strongly correlated systems.

## Abstract (web summary)

Quantum spin chains can be represented in several ways, and eigenstates in these dual models have different entanglement structures. By characterizing the dual theories, the optimal tensor network algorithm for simulating the models has been found.

## Main

Symmetry-breaking forms one of the cornerstones of many-body physics. This had been recognized by Pierre Curie in 1894, who wrote that dissymmetry creates all interesting phenomena^1,2^. Landau formalized this idea and suggested that different gapped symmetry-breaking phases could be characterized by local order parameters that transform non-trivially under the symmetry^3^. In 1971, Kadanoff and Ceva turned the tables and demonstrated that non-local order parameters can be used to characterize gapped symmetric phases^4^. For the Ising model, their non-local order parameter is obtained by applying the celebrated Kramers–Wannier duality transformation to the local one^5^. Such non-local order parameters play a prominent role in the characterization of topological phases of matter^6–9^: these phases cannot be distinguished from trivial phases by local order parameters, and therefore, they challenge the standard Landau paradigm.

The modern approach to characterizing phases of matter uses the language of entanglement and quantum circuits. Two gapped Hamiltonians with a given symmetry are in the same phase if and only if there exists a symmetry-preserving sublinear depth quantum circuit that transforms their ground states into each other^10,11^. In one dimension, it has been proven that there is an area law for the entanglement entropy for ground states of local gapped Hamiltonians^12^. Such states can be efficiently represented in terms of matrix product states (MPSs)^13^, whose (topological) phase can be characterized directly by the transformation properties of the entanglement degrees of freedom under the symmetry^9,14^. Importantly, the MPS description of a ground state can efficiently be obtained using the density matrix renormalization group (DMRG). These tensor-network-based algorithms effectively break down the exponential complexity wall for finding ground states of interacting one-dimensional quantum lattice models^15–17^. The less entanglement in the ground state, the better these algorithms perform.

Although traditional symmetry operators act in an on-site manner, tensor networks enable the definition of more general symmetries on the lattice that encode a correlated action on neighbouring sites. Mathematically, such generalized symmetries^18–28^ are described by a fusion category^29^, and they can be represented explicitly as matrix product operators (MPOs)^30–34^. The phases of such systems are classified by a choice of module category compatible with those symmetries^23,35–39^, and the generalized Landau paradigm^25,40–43^ entails that the inclusion of these generalized symmetries yields a complete classification of gapped phases. Crucially, module categories also classify the different ways a given (generalized) symmetry can be gauged^44–46^. The explicit operators representing those operations on the lattice are again of the MPO form^34^, and they map local order parameters to non-local ones, generalizing the aforementioned Kramers–Wannier duality. By allowing them to act on the boundary conditions, one can show that these can be lifted to unitary operations and, hence, preserve the full spectrum of the Hamiltonian^47,48^.

In this paper, we connect symmetry-breaking, duality and entanglement and present the following insight: for every Hamiltonian, one can determine an optimal dual Hamiltonian whose ground states minimize the amount of entanglement. More precisely, consider a Hamiltonian with a (generalized) symmetry and a ground state in a certain phase. By a (twisted) gauging of the remaining symmetries, the corresponding dual Hamiltonian will spontaneously break its dual symmetry completely. Doing so eliminates the degeneracy imposed by the symmetry on the entanglement spectrum, minimizing the entanglement compared to all other dual ground states. Additionally, a completely broken symmetry means that all variational parameters in the ground state are independent, leading to a large reduction in computational complexity. The ground states in the original theory can be obtained by multiplying the optimal dual ground states with duality operators in the form of an MPO^34,47^, which reintroduces the multiplicities in the entanglement spectra and enlarges the bond dimension. For twisted gauging, this procedure maps symmetry-protected topological phases to trivial symmetry-broken ones and vice versa.

The only price we pay by considering a dual model is that it may not be defined on a tensor product Hilbert space due to kinematical constraints introduced by the gauging procedure. We overcome this by developing a variant of the DMRG algorithm that directly incorporates these constraints, and we demonstrate that all the building blocks for state-of-the-art implementations of tensor networks algorithms are still in place. When applying our method to an ordinary symmetric phase, the variational parameters are effectively the same as in symmetric tensor network methods^49–51^. However, by defining Hamiltonians directly in the dual space^34,47^, our method yields a much simpler way of optimizing over those parameters, and furthermore, it allows us to extend these methods to work in any phase.

## Results

### Hamiltonian and gapped phases

Before introducing the general framework, we demonstrate our formalism with a Heisenberg-like model representing various gapped phases. Although this rather simple model hosts a conventional on-site symmetry, the optimal dual models exhibit generalized symmetries, demonstrating their importance in computational methods. While we illustrate our approach for a two-site nearest-neighbour Hamiltonian, our formalism readily generalizes to longer-range Hamiltonians as well as to transfer matrices of classical statistical mechanical models.

Consider an open quantum spin chain of length *L* and assign to every site i spin-1 degrees of freedom with spin operators $({S}_{{\mathsf{i}}}^{x},{S}_{{\mathsf{i}}}^{y},{S}_{{\mathsf{i}}}^{z})$. The dynamics is governed by the following family of Hamiltonians^52^:

1 $${\mathbb{H}}=\sum_{{\mathsf{i}}=1}^{L-1}\left({{\mathbb{h}}}_{{\mathsf{i}},0}+{J}_{1}{{\mathbb{h}}}_{{\mathsf{i}},1}+{J}_{2}{{\mathbb{h}}}_{{\mathsf{i}},2}\right),$$

with coupling constants *J*_1_ and *J*_2_ and local operators

$$\begin{aligned}{{\mathbb{h}}}_{{\mathsf{i}},0}&:={S}_{{\mathsf{i}}}^{x}{S}_{{\mathsf{i}}+1}^{x}+{S}_{{\mathsf{i}}}^{y}{S}_{{\mathsf{i}}+1}^{y}+{S}_{{\mathsf{i}}}^{z}{S}_{{\mathsf{i}}+1}^{z},\\ {{\mathbb{h}}}_{{\mathsf{i}},1}&:={({S}_{{\mathsf{i}}}^{x}{S}_{{\mathsf{i}}+1}^{x})}^{2}+{({S}_{{\mathsf{i}}}^{y}{S}_{{\mathsf{i}}+1}^{y})}^{2}+{({S}_{{\mathsf{i}}}^{z}{S}_{{\mathsf{i}}+1}^{z})}^{2},\\ {{\mathbb{h}}}_{{\mathsf{i}},2}&:=\{{S}_{{\mathsf{i}}}^{x},{S}_{{\mathsf{i}}}^{y}\}{S}_{{\mathsf{i}}+1}^{z}+\{{S}_{{\mathsf{i}}}^{z},{S}_{{\mathsf{i}}}^{x}\}{S}_{{\mathsf{i}}+1}^{y}+\{{S}_{{\mathsf{i}}}^{y},{S}_{{\mathsf{i}}}^{z}\}{S}_{{\mathsf{i}}+1}^{x}.\end{aligned}$$

Although the term ${{\mathbb{h}}}_{{\mathsf{i}},0}$ defines the spin-1 antiferromagnetic Heisenberg model, which is SO(3) symmetric, the terms ${{\mathbb{h}}}_{{\mathsf{i}},1}$ and ${{\mathbb{h}}}_{{\mathsf{i}},2}$ are perturbations breaking the symmetry down to the finite subgroup ${{\mathbb{A}}}_{4}\subset {\rm{SO}}(3)$ of orientation-preserving symmetries of the tetrahedron. The spin-1 degrees of freedom transform as the three-dimensional representation of ${{\mathbb{A}}}_{4}$, which we denote by $\underline{3}$.

In the presence of a symmetry ${{\mathbb{A}}}_{4}$, we distinguish seven possible gapped phases. Each gapped phase is labelled by a subgroup $H\subseteq {{\mathbb{A}}}_{4}$ together with a class [*ψ*] in the second cohomology group of *H*, which classifies its projective representations. Physically, although *H* characterizes the symmetry preserved within the ground-state subspace, a non-trivial [*ψ*] signals the presence of edge modes that transform projectively under the action of the subsymmetry *H* (ref. ^9^). Up to isomorphisms, ${{\mathbb{A}}}_{4}$ has five subgroups, namely ${{\mathbb{A}}}_{4}$, ${{\mathbb{D}}}_{2}$, ${{\mathbb{Z}}}_{3}$, ${{\mathbb{Z}}}_{2}$ as well as the trivial one. Out of these subgroups, only ${{\mathbb{A}}}_{4}$ and ${{\mathbb{D}}}_{2}$ have non-trivial second cohomology groups, which are both isomorphic to ${{\mathbb{Z}}}_{2}$. Hence, there are seven gapped phases.

### Dual ground states as MPSs

Following refs. ^34,47^, Hamiltonians dual to equation (1) can be obtained by acting with a duality operator ${\mathbb{D}}$ on the local ${{\mathbb{A}}}_{4}$ symmetric terms of the Hamiltonian as

2 $${{\mathbb{h}}}_{{\mathsf{i}},n}^{\text{dual}}\circ {\mathbb{D}}={\mathbb{D}}\circ {{\mathbb{h}}}_{{\mathsf{i}},n},\quad n\in \{0,1,2\}.$$

These operators preserve the algebra generated by the local symmetric operators, and thus, they preserve the spectrum of the Hamiltonian up to degeneracies^47,48^. In particular, ground state(s) of the original Hamiltonian can be obtained from ground state(s) of any dual model as $\left\vert {\psi }_{{\rm{g.s.}}}\right\rangle ={{\mathbb{D}}}^{\dagger }\left\vert {\psi }_{\,\text{g.s.}}^{\text{dual}\,}\right\rangle$. Note that these dualities typically relate models whose ground-state subspaces have different dimensions. By carefully considering the action of the duality on boundary conditions—and, if necessary, introducing ancillary degrees of freedom—these duality transformations can be lifted to unitary matrices^34,48^.

The list of possible duality operators ${\mathbb{D}}$ at our disposal (and, thus, the list of possible dual Hamiltonians ${{\mathbb{H}}}^{{\rm{dual}}}$) is completely determined by the symmetry of the original model. For the symmetry group ${{\mathbb{A}}}_{4}$, the various dual models are labelled by a choice of subgroup $H\subseteq {{\mathbb{A}}}_{4}$ together with a class [*ψ*] in the second cohomology group of *H*, which matches the classification of possible gapped phases with a total of seven distinct dual Hamiltonians. The duality associated with the pair (*H*, [*ψ*]) amounts to the *ψ*-twisted gauging of the subgroup *H* (refs. ^45,46^). This gauging procedure requires the introduction of gauge degrees of freedom, which are labelled by irreducible projective representations of *H* with respect to *ψ* on the links between neighbouring sites. These projective representations and their intertwiners are organized into an algebraic structure denoted by ${{\mathsf{Rep}}}^{\psi }(H)$. Moreover, the gauge degrees of freedom are required to satisfy local Gauss constraints, so that dual models typically act on a Hilbert space that is not a tensor product space.

The ground states for these dual models can be parametrized as MPSs, which are states of the form

$$\left\vert {\psi }_{\text{g.s.}}^{\text{dual}}\right\rangle =\sum_{{i}_{1},\ldots ,{i}_{L}}{A}_{1}^{{i}_{1}}{A}_{2}^{{i}_{2}}\cdots {A}_{L}^{{i}_{L}}\left\vert {i}_{1}{i}_{2}\ldots {i}_{L}\right\rangle.$$

with each ${A}_{{\mathsf{i}}}^{i}$ a ${\chi }_{{\mathsf{i}}-1}\times {\chi }_{{\mathsf{i}}}$ matrix. The various ${\chi }_{{\mathsf{i}}}$ are referred to as bond dimensions and are such that *χ*_0_ = *χ*_*L*_ = 1. Because these ground states satisfy an area law for the entanglement entropy, the bond dimensions ${\chi }_{{\mathsf{i}}}$ that characterize the amount of entanglement between neighbouring sites do not scale with the system size *L*. These MPSs live in a Hilbert space where the physical ${{\mathsf{Rep}}}^{\psi }(H)$-labelled gauge degrees of freedom on the links are duplicated to the two corresponding vertices, so that the Gauss constraints can be imposed at the vertices. This implies that every virtual bond index of the MPS carries another label of the corresponding gauge degree of freedom, and this allows us to label the entanglement spectrum with labels in ${{\mathsf{Rep}}}^{\psi }(H)$. To compute these MPS ground states, we use a generalized DMRG algorithm that manifestly preserves the constraints in the Hilbert space (Methods). Once we obtain the ground state of a dual model, it can be transformed to a ground state of the original model using the corresponding duality operator, which can itself be parametrized as an MPO of the form

$${\mathbb{D}}=\sum_{\begin{array}{c}{i}_{1},\ldots ,{i}_{L}\\ {j}_{1},\ldots ,{j}_{L}\end{array}}{D}^{{i}_{1}{j}_{1}}{D}^{{i}_{2}{j}_{2}}\cdots {D}^{{i}_{L}{j}_{L}}\left\vert{i}_{1}{i}_{2}\ldots {i}_{L}\right\rangle \left\langle{j}_{1}{j}_{2}\ldots {j}_{L}\right\vert ,$$

with each *D*^*ij*^ a *χ* × *χ* matrix. The uncontracted matrix indices on sites 1 and *L* correspond to the different boundary conditions for the duality MPO, which are the extra degrees of freedom required to make these duality operators unitary. Because this MPO itself has a non-trivial bond dimension *χ*, its action onto an MPS generically yields an MPS of larger bond dimension with more entanglement.

### Numerical results

**Fig. 1:**
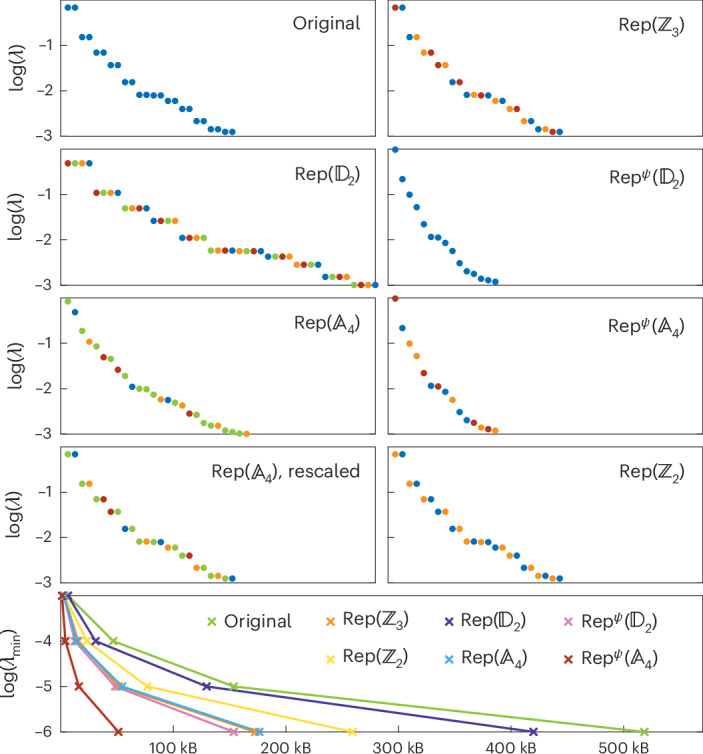
${\pmb{\mathbb{A}}}_{\mathbf{4}}$ SPT phase. Top: entanglement spectra of the dual models in the middle of the ground state for 60 sites in the ${{\mathbb{A}}}_{4}$ SPT phase of the initial model (*J*_1_ = 1 and *J*_2_ = 1). The colour of the Schmidt value indicates the object that labels the corresponding gauge degree of freedom. Bottom: memory required to store a ground-state MPS tensor in the bulk at double precision for a given truncation error *λ*_min_. The ground state of the ${{\mathsf{Rep}}}^{\psi }({{\mathbb{A}}}_{4})$ model minimizes the entanglement and the number of variational parameters for a fixed truncation error. Source data

**Fig. 2:**
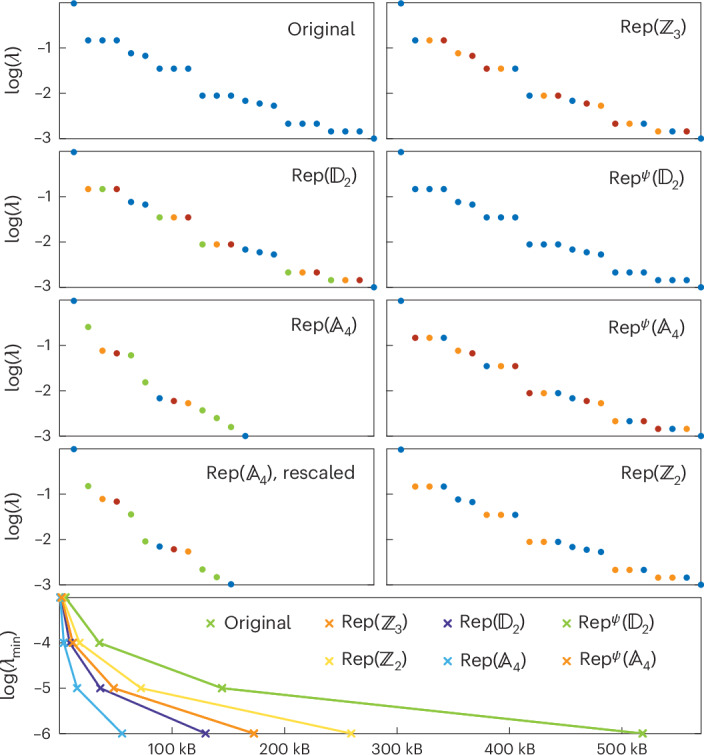
${\pmb{\mathbb{A}}}_{\mathbf{4}}$ symmetric phase. Top: entanglement spectra of the dual models in the middle of the ground state for 60 sites in the ${{\mathbb{A}}}_{4}$ symmetric phase of the original model (*J*_1_ = −2 and *J*_2_ = −5). The colour of a Schmidt value indicates the object that labels the corresponding gauge degree of freedom. Bottom: memory required to store a ground-state MPS tensor in the bulk at double precision for a given truncation error *λ*_min_. The ground state of the ${\mathsf{Rep}}({{\mathbb{A}}}_{4})$ model minimizes the entanglement and number of variational parameters for a fixed truncation error. Source data

**Fig. 3:**
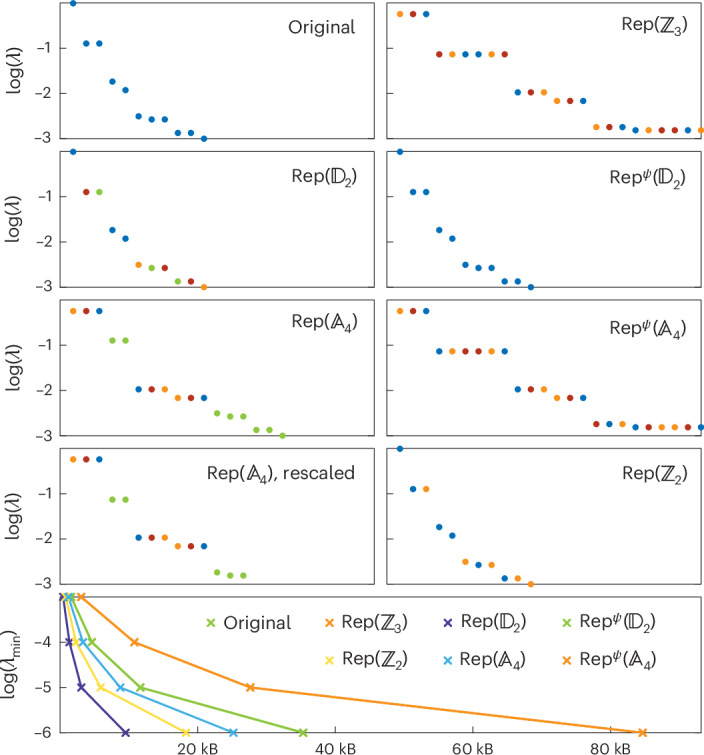
${{\mathbb{D}}}_{\mathbf{2}}$ symmetric phase. Top: entanglement spectra of the dual models in the middle of the ground state for 60 sites in the ${{\mathbb{D}}}_{2}$ symmetric phase of the original model (*J*_1_ = −5 and *J*_2_ = 1). The colour of a Schmidt value indicates the object that labels the corresponding gauge degree of freedom. Bottom: memory required to store a ground-state MPS tensor in the bulk at double precision for a given truncation error *λ*_min_. The ground state of the ${\mathsf{Rep}}({{\mathbb{D}}}_{2})$ model minimizes the entanglement and the number of variational parameters for a fixed truncation error. Source data

We consider points (*J*_1_, *J*_2_) = {(1, 1), (−2, −5), (−5, 1)} in the phase diagram of the model of equation (1) that represent the ${{\mathbb{A}}}_{4}$ symmetry-protected topological (SPT) phase, the ${{\mathbb{A}}}_{4}$ symmetric phase and the ${{\mathbb{D}}}_{2}$ symmetric phase, respectively. For each point, we simulate all seven dual Hamiltonians labelled by ${{\mathsf{Rep}}}^{\psi }(H)$, which also includes the original one. For the three phases, we plot the entanglement spectra of the dual ground states coloured by the different objects labelling the gauge degrees of freedom as well as the memory requirements to reach a specific minimal Schmidt value *λ*_min_ that serves as a proxy error measure. Our findings are displayed in Figs. 1–3 and analysed below.

#### ${\pmb{\mathbb{A}}}_{\mathbf{4}}$ SPT phase

In this phase (Fig. 1), the entanglement degrees of freedom of the unique ground state transform as projective representations of ${\mathbb{A}}_{4}$. The three irreducible projective representations of ${{\mathbb{A}}}_{4}$ are two-dimensional, which explains the twofold degeneracy for every Schmidt value. Comparing the entanglement spectra of the various dual models, we observe that the entanglement is minimized in the ${{\mathsf{Rep}}}^{\psi }({{\mathbb{D}}}_{2})$ and ${{\mathsf{Rep}}}^{\psi }({{\mathbb{A}}}_{4})$ models. Additionally, the ground state of the ${{\mathsf{Rep}}}^{\psi }({{\mathbb{A}}}_{4})$ model requires the least amount of memory, making this model the most efficient one to simulate. Crucially, this dual model possesses a non-invertible ${\mathsf{Rep}}({{\mathbb{A}}}_{4})$ symmetry, whereby symmetry operators are labelled by representations of ${{\mathbb{A}}}_{4}$, which is completely broken in the ground-state subspace. An important subtlety with a non-invertible symmetry-breaking phase is that the different ground states, being related by non-trivial MPOs, can have different entanglement properties, making them distinguishable. In this particular example, the ground state labelled by the three-dimensional irreducible representation requires more entanglement and therefore more variational parameters than the other ground states. In practice, it is possible to avoid this ground state by biasing the initial MPS towards the other less entangled ground states.

All the other dual models admit ground states that preserve some symmetry, resulting in these cases in degeneracy in the entanglement spectrum as well excessive memory requirements. This is ostensible in the ${\mathsf{Rep}}({{\mathbb{D}}}_{2})$ model, where every Schmidt value has a fourfold degeneracy due to its ground state preserving a dual ${{\mathbb{A}}}_{4}$ symmetry, the ${{\mathbb{D}}}_{2}$ subgroup of which permutes the gauge degrees of freedom that label the Schmidt values, which happen to be labelled by irreducible representations of ${{\mathbb{D}}}_{2}$. A more subtle case is the ${\mathsf{Rep}}({{\mathbb{A}}}_{4})$ model, which has a non-invertible ${\mathsf{Rep}}({{\mathbb{A}}}_{4})$ symmetry that is only partially broken in the ground-state subspace. Although the remaining symmetry constrains the entanglement spectrum, the degeneracy is hidden as the ratios between consecutive Schmidt values labelled by the three- and one-dimensional irreducible representation are fixed to $\sqrt{3}$. We make this manifest by rescaling the spectrum appropriately.

#### ${\pmb{\mathbb{A}}}_{\mathbf{4}}$ symmetric phase

In this phase (Fig. 2), the entanglement degrees of freedom of the unique ground state transform as linear representations of ${{\mathbb{A}}}_{4}$, the three-dimensional irreducible representation $\underline{3}$ explaining occurrences of threefold degeneracy in the entanglement spectrum. Comparing the entanglement spectra of the various dual models, we observe that the entanglement is minimized in the ${\mathsf{Rep}}({{\mathbb{A}}}_{4})$ model. Additionally, the ground state of this model requires the least amount of memory, making it the most efficient one to simulate. As for the previous phase, this optimal dual model has a non-invertible symmetry ${\mathsf{Rep}}({{\mathbb{A}}}_{4})$, which also happens to be completely broken in the ground-state subspace. Finding the ground state of this optimal dual model is effectively what existing symmetric tensor network methods achieve (Methods).

All the other dual models admit ground states sharing the same entanglement spectrum as the initial model, differing only in the labelling of the Schmidt values and the improvement in the memory requirements. These dual models possess either invertible or non-invertible symmetries, and the ground states break various amounts thereof, but the whole symmetry is never broken. To verify the absence of hidden degeneracies, we have rescaled the entanglement spectrum of the ${\mathsf{Rep}}({{\mathbb{A}}}_{4})$ model.

#### ${\mathbb{D}}_{2}$ symmetric phase

In this phase (Fig. 3), the entanglement degrees of freedom of the ground states transform as linear representations of ${{\mathbb{D}}}_{2}$. Because irreducible representations of ${{\mathbb{D}}}_{2}$ are all one-dimensional, no other degeneracy in the entanglement spectrum is enforced. The visible twofold degeneracies must originate from a hidden symmetry that might involve time reversal combined with a physical on-site action. Comparing the entanglement spectra of the various dual models, we observe that the entanglement is minimized in the initial model, as well as the ${{\mathsf{Rep}}}^{\psi }({{\mathbb{D}}}_{2})$, ${\mathsf{Rep}}({{\mathbb{D}}}_{2})$ and ${\mathsf{Rep}}({{\mathbb{Z}}}_{2})$ models. However, the ${\mathsf{Rep}}({{\mathbb{D}}}_{2})$ model stands out as requiring the least amount of memory, making this model the most efficient one to simulate. This optimal dual model has an ${{\mathbb{A}}}_{4}$ symmetry, which happens to be completely broken in the ground-state subspace.

The remaining dual models admit ground states showing more entanglement, as a consequence of Schmidt values in the initial ground state becoming degenerate. This is most easily understood in the ${\mathsf{Rep}}({{\mathbb{Z}}}_{3})$ model that possesses a non-invertible ${\mathsf{Rep}}({{\mathbb{A}}}_{4})$, which is fully preserved by the unique ground state. The one-dimensional irreducible representations act by permuting gauge degrees of freedom labelled by irreducible representations of ${{\mathbb{Z}}}_{3}$, thus enforcing further threefold degeneracy. A similar explanation holds for the degenerate Schmidt values in the ${\mathsf{Rep}}({{\mathbb{A}}}_{4})$ and ${{\mathsf{Rep}}}^{\psi }({{\mathbb{A}}}_{4})$ models. To verify the absence of hidden degeneracies, we have rescaled the entanglement spectrum of the ${\mathsf{Rep}}({{\mathbb{A}}}_{4})$ model.

In all the above examples, the dual model that is the most computationally efficient to simulate in terms of memory requirements is always the unique one whose dual symmetry is completely broken in the ground-state subspace. This is the model obtained by performing the (possibly) twisted gauging of the symmetry that is preserved within the ground-state subspace of the initial model. Performing the ground-state computation in the specific dual model obtained by gauging the full symmetry of the Hamiltonian is effectively equivalent (Methods) to the approach taken by symmetric tensor network methods^49–51^. As we have seen, however, this is only optimal in the symmetric phase, and imposing too much symmetry requires further long-range entanglement, which adversely affects the performance of the algorithms. By going to the optimal dual model where all symmetry is broken, our methods do not suffer from this and outperform the current state-of-the-art algorithms, for both memory and computational time requirements. Additionally, they are much simpler to implement and uncover the mathematical structure underlying the quasiparticle excitations, as we argue in the next section.

### General framework

The results presented above hold much more broadly. Consider a generalized symmetry in a one-dimensional quantum lattice model, that is, a symmetry whose operators are not necessarily invertible^20–25,27,28,45^. The symmetry operators take the form of (typically non-local) MPOs^31–34,47^. Mathematically, a finite generalized symmetry can be modelled by a so-called fusion category^53^, which extends the group theoretical formalism of ordinary symmetries. Like ordinary symmetries, generalized symmetries can be spontaneously broken—as we already witnessed in our examples—and may be gauged provided that there is no ’t Hooft anomaly. These possible gaugings are classified by a choice of module category over the symmetry fusion category, which is the same classification as the possible gapped phases with respect to such a symmetry^22,23,36,38,54,55^.

Consider a one-dimensional quantum lattice model with a generalized symmetry. Suppose the symmetry is completely broken in the ground-state subspace. By gauging the symmetry, which amounts to identifying the corresponding symmetry operators as well as the corresponding symmetry-broken states, we obtain a dual model with a dual symmetry that is fully preserved by the unique ground state. Crucially, gauging this dual symmetry recovers the initial model. More generally, there is always a way to gauge the (sub)symmetry that is preserved in the ground-state subspace of a model so as to yield a dual model whose dual generalized symmetry is completely broken (Methods). In practice, this dual model is obtained by extending the approach followed in our series of examples. First, we write the Hamiltonian in terms of symmetric tensors that make the generalized symmetry of the model manifest. Then, these symmetric tensors are replaced by dual symmetric tensors, which correspond to the relevant gauging of the preserved subsymmetry, yielding the dual model (Methods).

We claim that the optimal way of simulating the phase of a given model amounts to simulating the dual phase of this dual model where the dual symmetry is completely broken, after which we recover the original ground state by acting with the MPO that transmutes the corresponding Hamiltonians into each other. Broadly speaking, the reasoning is that any symmetry translates into constraints among the variational parameters so that they are not all independent. The associated redundancy unequivocally translates into a suboptimal use of computational resources, as we observed in the examples above.

**Fig. 4:**
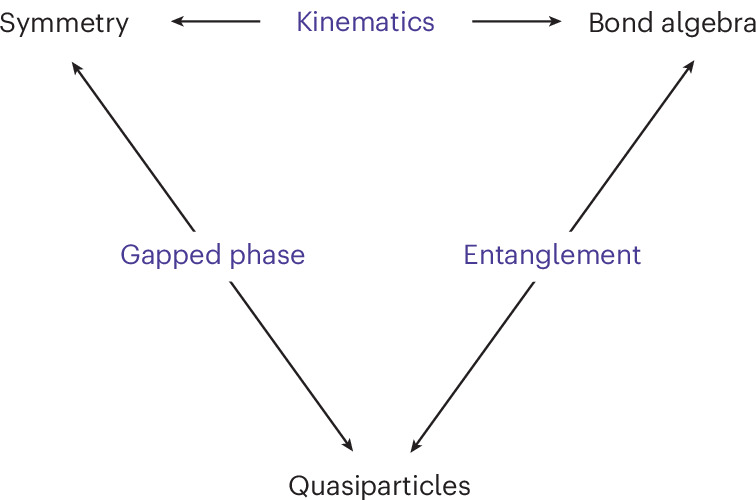
Categorical structures. The arrows denote relations between the fusion categories that organize the symmetry, the bond algebra generated by the Hamiltonian terms and the quasiparticle excitations. Given an abstract bond algebra of symmetric operators, there are different choices for the kinematical degrees of freedom on which it can be represented. A particular choice then determines the explicit Hamiltonian and, subsequently, its symmetries. Similarly, given a symmetry, there are different gapped phases that a system with such a symmetry can exhibit. A particular choice of phase then determines what the possible quasiparticles are, for example, domain-wall or charge excitations. This diagram means that the composition of these relations is consistent in the sense that the kinematical degrees of freedom together with the phase of the model uniquely specify the structure of the edge modes or, equivalently, the entanglement degrees of freedom. We explain this diagram in more detail in Methods.

In the optimal dual phase, at least one of the ground states has the property that the action of any dual symmetry operator on it yields an orthogonal ground state. In this phase, the ground states are in one-to-one correspondence with symmetry operators, and the state having this property is that corresponding to the identity operator. For this maximal symmetry-breaking state, all order parameters are strictly local. This follows because the action of the MPOs representing the dual symmetries map such a ground state into a different one, and, hence, the expectation value of any non-local string order operator vanishes exponentially in the number of sites on which it acts^56^. Conversely, all quasiparticle excitations on top of this maximal symmetry-breaking ground state correspond to domain-wall excitations. For an infinite (1 + 1)-dimensional lattice model, these excitations are created by the action of the symmetry MPOs on one half of the chain^57^. As in the usual ansatz for topological excitations in MPS, further variational degrees of freedom characterizing the precise nature of the excitations emerge at the end point of the MPO^58,59^. The domain-wall excitations of the dual symmetry-breaking model are mapped to the quasiparticle excitations of the original model, which can be of a very different nature (spinon, holon and so on). However, the equivalence between dual theories implies that the fusion category describing the quasiparticle excitations remains the same, thus providing a characterization of these excitations in any possible gapped phase. Our work reveals the underlying relations between the category theoretic structures describing the properties of a gapped ground state of a (generalized) symmetric Hamiltonian in (1 + 1) dimensions. Graphically, these relations are summarized in Fig. 4.

## Discussion

Although fusion categories deal with a finite number of objects, our formalism is more general and also works for continuous symmetries described by Lie groups. In particular, when dealing with a model with an on-site SU(2) symmetry in the symmetric phase, the required duality MPO boils down to the interaction-round-a-face-vertex transformation^60,61^, which is a special case of the more general Schur–Weyl duality for SU(*N*). At present, our algorithm takes advantage of only internal symmetries, because it exploits an approach to dualities that has been tailored to this type of symmetry. It will be very interesting to generalize this approach to different types of symmetry, such as spatial symmetries and time reversal, which would lead to further refinements of phase diagrams.

Although we have restricted the analysis to gapped phases, we expect that our approach will also be useful for studying phase transitions between gapped phases. At these critical points, the ground state no longer admits a description in terms of an MPS with finite bond dimension, and working with such an MPS induces a relevant perturbation to the critical Hamiltonian. By increasing the bond dimension, the strength of this relevant perturbation decreases, and one can use this to extrapolate and obtain accurate predictions for critical exponents; this is known as entanglement scaling^62,63^. The nature of the relevant perturbation depends strongly on the symmetries present in the MPS, and by using different dual models, we are effectively approaching the critical point from different directions. By combining the entanglement scaling results from these different dual models, we expect to realize an improvement in the accuracy of the predicted critical exponents.

One of the main merits of our method is that it is systematically extensible to higher-dimensional models and to tensor network algorithms in terms of projected entangled pair states^64^. In fact, taking advantage of symmetries in higher dimensions is expected to be even more beneficial than in (1 + 1) dimensions. Although formally more challenging, many aspects of dualities in two-dimensional quantum lattice models have been worked out^65^. Specifically, the relevant tensor network operators are already known for a large class of generalized symmetries^66–68^. In particular, models in (2 + 1) dimensions with 1-form symmetries admit tensor network representations where these symmetries manifest themselves on the entanglement degrees of freedom and are, therefore, robust under perturbations such that they also capture models where the physical higher form symmetry is emergent^56^. These virtual symmetries become physical in the double-layer transfer matrix, which can then be exploited in the computation of the (1 + 1) dimensions environment using the methods in this work. We expect this type of dimensional reduction to hold in higher dimensions as well, where the computational gains are even more pronounced.

## Methods

### Dualities

We explain here how to obtain the models dual to equation (1) considered in the main text. We encourage the reader to consult ref. ^48^ for further details. First, it is crucial to rewrite the local operators entering the definition of the Hamiltonian of equation (1) in such a way that the symmetry ${{\mathbb{A}}}_{4}$ is manifest. Invoking a finite group version of the Wigner–Eckart theorem, we know that any operator transforming trivially under ${{\mathbb{A}}}_{4}$ must be expressible as a linear combination of Clebsch–Gordan coefficients. The group ${{\mathbb{A}}}_{4}$ possesses three one-dimensional irreducible representations $\{\underline{0},\underline{1},{\underline{1}}^{* }\}$ and a single three-dimensional one $\underline{3}$ satisfying $\underline{3}\otimes \underline{3}\cong \underline{0}\oplus \underline{1}\oplus {\underline{1}}^{* }\oplus \underline{3} \oplus \underline{3}$. Given three irreducible representations *V*_1_, *V*_2_ and *V*_3_ such that *V*_3_ ⊂ *V*_1_ ⊗ *V*_2_, we interpret the intertwining map *V*_3_ → *V*_1_ ⊗ *V*_2_ as the tensorwhere the sums are over basis vectors and *i* enumerates the different ways *V*_1_ ⊗ *V*_2_ decomposes into *V*_3_. In this equation, the diagram on the right-hand side depicts the Clebsch–Gordan coefficients valued in ${\mathbb{C}}$. In this notation, we can show that the local operators ${{\mathbb{h}}}_{{\mathsf{i}},n}$, *n* ∈ {0, 1, 2}, are all of the form3where ${h}_{n}(V,i,j)\in {\mathbb{C}}$. Notice that in this formulation, the state space of a given spin-1 degree of freedom is spanned by $\left\vert \underline{3},v\right\rangle$, with *v* = 1, …, 3.

Importantly, we have the following equality of intertwining maps *V*_4_ → *V*_1_ ⊗ *V*_2_ ⊗ *V*_3_:2where the *F* symbols ${\left({F}_{{V}_{4}}^{{V}_{1}{V}_{2}{V}_{3}}\right)}_{{V}_{5},ij}^{{V}_{6},kl}\in {\mathbb{C}}$ are provided by the Racah *W* coefficients of ${{\mathbb{A}}}_{4}$. One can use this identity to show that the structure constants of the algebra generated by the local symmetric operators ${\{{{\mathbb{h}}}_{{\mathsf{i}},n}\}}_{{\mathsf{i}},n}$ depend only on the *F* symbols and coefficients ${\{{h}_{n}\}}_{n}$. Interpreting equation (4) as a tensor network equation, one can ask the following question: Is there another set of tensors, generically depicted as56also indexed by triplets of representations {*V*_1_, *V*_2_, *V*_3_ ⊂ *V*_1_ ⊗ *V*_2_} and satisfying equation (4)? Keeping ${\{{h}_{n}\}}_{n}$ the same, then replacing the intertwining maps in equation (3) by these new tensors would result in an isomorphic algebra of local operators and, thus, a dual model with Hamiltonian^34^which can be checked to have the same spectrum^47^. When dealing with a symmetry ${{\mathbb{A}}}_{4}$, one can find collections of tensors of the form of equation (5) satisfying equation (4) for any $H\subseteq {{\mathbb{A}}}_{4}$ and [*ψ*] ∈ *H*^2^(*H*, U(1)) (ref. ^69^). This is because dualities correspond to (twisted) gauging maps of the symmetry ${{\mathbb{A}}}_{4}$, and there are as many ways to gauge a symmetry as there are ways to spontaneously break it. Typically, the dual Hamiltonian acts on a distinct microscopic Hilbert space, which is not necessarily a tensor product space as suggested by the graphical notation. This is consistent with the gauging interpretation, leading to theories with gauge degrees of freedom that satisfy local Gauss constraints. Given a pair (*H*, [*ψ*]), the degrees of freedom of the resulting dual model are associated with irreducible *ψ*-projective representations^34^, and as such, we label the model by ${{\mathsf{Rep}}}^{\psi }(H)$. Instead of Clebsch–Gordan coefficients, the tensors are found to evaluate to Racah *W* coefficients involving linear representations of *G* and *ψ*-projective representations of the subgroup *H*. Crucially, the initial Hamiltonian can be transmuted into any of its duals through an MPO^47^:7which is true for any *V*, *i* and *j*.

### Generalized DMRG

To compute the ground states of the various models in the main text, we implemented a version of the two-site DMRG algorithm for finite chains with open boundary conditions^70^. The DMRG algorithm is a variational algorithm within the subspace of MPSs, which we recall is a class of wavefunctions that implement the area law of gapped phases at the microscopic level, thereby specifically targeting the physical corner of the total Hilbert space. Briefly, the DMRG algorithm proceeds as follows. Because the wavefunction is a multilinear function of the variables in all local tensors, the global optimization problem can iteratively be solved using an alternating least-squares approach^71^. The two-site version proceeds by blocking two sites together before solving the combined least-squares problem and finally using a singular value decomposition to split the two-site tensor into two one-site tensors. This two-site version is typically preferred, as it allows for an easier redistribution of Schmidt coefficients in the different tensor blocks.

Typically, MPSs are taken to span a subspace of a tensor product space. However, an important feature of the models we consider is that they are typically not defined on a tensor product space. Rather, states need to satisfy some local kinematical constraints; these are the local Gauss constraints mentioned above. Thus, we require MPSs that explicitly enforce these kinematical constraints. For our illustrative example, this is accomplished by considering tensors of the form8where *V*_1_ and *V*_2_ are summed over *ψ*-projective irreducible representations of some subgroup $H\subseteq {{\mathbb{A}}}_{4}$, *i* over basis vectors in the space of intertwining maps ${V}_{1}\otimes \underline{3}\to {V}_{2}$, and *d*_1_ and *d*_2_ label the remaining variational degrees of freedom. Notice that both entanglement degrees of freedom labelled by (*V*, *d*) and physical degrees of freedom labelled by $({V}_{1}\underline{3}{V}_{2},i)$ carry gauge degrees of freedom represented by blue lines, which are shared by neighbouring physical degrees of freedom, as indicated by our graphical notation. For instance, the local Hilbert space on two sites is spanned by vectors of the form $\left\vert {V}_{1}\underline{3}{V}_{2},i\right\rangle \left\vert {V}_{2}\underline{3}{V}_{3},j\right\rangle$. Typically, the dimension of the space of intertwining maps ${V}_{1}\otimes \underline{3}\to {V}_{2}$ depends on (*V*_1_, *V*_2_), which is incompatible with a tensor product Hilbert space. By construction, the action of the Hamiltonian of equation (6) leaves the constrained Hilbert space invariant and, thus, explicitly preserves the structure of such an MPS.

Our algorithm proceeds like the standard two-site DMRG, but all the basic operations are tailored to preserve the kinematical constraints, which amounts to maintaining the structure displayed in equation (8). First, by using block-diagonal basis transformations on the entanglement space, any MPS of the form of equation (8) can be brought into the left canonical form defined by the condition:9In left canonical form, the Schmidt values *λ* of the reduced density matrix obtained by tracing out all the sites to the right of the site ${\mathsf{i}}$ are then given by the spectrum of ${\rho }_{{\mathsf{i}}}$ defined as10These density matrices are block diagonal, with blocks labelled by *ψ*-projective irreducible representations of the subgroup *H*. As reviewed above, a crucial step of the two-site DMRG algorithm amounts to decomposing the two-site MPS tensor that solves the combined least-squares problem into single-site MPS tensors *A*. Specifically, consider a tensor whose entries are of the form11Keeping the gauge degree of freedom *V*_2_ fixed, one considers the matrix ${B}^{{V}_{2}}$ with entries

12 $${[{B}^{{V}_{2}}]}_{({V}_{1},{d}_{1}{i}_{1})}^{({V}_{3},{d}_{3}{i}_{2})}:={[{B}^{({V}_{1}\underline{3}{V}_{2},{i}_{1})({V}_{2}\underline{3}{V}_{3},{i}_{2})}]}_{({V}_{1},{d}_{1})}^{({V}_{3},{d}_{3})}.$$

Performing a singular value decomposition yields a factorization of the form ${B}^{{V}_{2}}={M}^{{V}_{2}}{\Sigma }^{{V}_{2}}{({N}^{{V}_{2}})}^{\dagger }$, where *M* and *N* are unitary matrices and ${\Sigma }^{{V}_{2}}$ is a diagonal matrix. The entries of *Σ* are all positive and are referred to as the singular values of ${B}^{{V}_{2}}$. Truncating the singular values to the desired precision *λ*_min_ yields the low-rank approximation

$${[{B}^{{V}_{2}}]}_{({V}_{1},{d}_{1}{i}_{1})}^{({V}_{3},{d}_{3}{i}_{2})}\approx\sum_{k=1}^{{\lambda }_{\min }}{[{M}^{{V}_{2}}]}_{({V}_{1},{d}_{1}{i}_{1})}^{k}\,{[{\Sigma }^{{V}_{2}}]}_{k}^{k}\,{{[{N}^{{V}_{2}}]}_{({V}_{3},{d}_{3}{i}_{2})}^{k}}^{* }.$$

Repeating this operation for all *ψ*-projective representations *V*_2_ of *A*, one finally defines MPS tensors ${A}_{{\mathsf{i}}}$ and ${A}_{{\mathsf{i}}+1}$:

13 $$\begin{aligned}{[{A}_{{\mathsf{i}}}^{({V}_{1}\underline{3}{V}_{2},{i}_{1})}]}_{({V}_{1},{d}_{1})}^{({V}_{2},k)}&:={[{M}^{{V}_{2}}]}_{({V}_{1},{d}_{1}{i}_{1})}^{k}\\ \text{and}\quad {[{A}_{{\mathsf{i}}+1}^{({V}_{2}\underline{3}{V}_{3},{i}_{2})}]}_{({V}_{2},k)}^{({V}_{3},{d}_{3})}&:={[{\Sigma }^{{V}_{2}}]}_{k}^{k}\,{{[{N}^{{V}_{2}}]}_{({V}_{3},{d}_{3}{i}_{2})}^{k}}^{* },\end{aligned}$$

respectively, so that equation (11) is approximated by14We can then repeat the same steps for the sites ${\mathsf{i}}+1$ and ${\mathsf{i}}+2$ and keep on sweeping from left to right and then from right to left.

In our simulations, we initialized the bulk of the MPS with random matrices of a given dimension per block *V*_2_, whereas on the boundary, we imposed a restriction to a single one-dimensional block. This corresponds to a choice of boundary condition for the MPO transmuting the Hamiltonian of the model that we are simulating into the initial one. In the very specific cases where our algorithm is equivalent to the standard symmetry-preserving DMRG, this amounts to the customary fixing of the total charge sector of the state. Finally, note that our approach is not specific to the two-site DMRG. In particular, uniform MPS algorithms for infinite chains (including variational uniform MPS (VUMPS) and ‘pulling-through’ algorithms) can also be implemented^72,73^.

### Symmetries in tensor networks

Next, we clarify the specific scenarios in which our algorithm amounts to using symmetry-preserving tensors. Consider a Hamiltonian with an ordinary symmetry encoded into a (finite) group *G*. Suppose the Hamiltonian is in the *G* symmetric phase. We claim that the optimal way of simulating this phase through the DMRG algorithm is to simulate the dual model obtained by gauging *G*, before acting with the MPO transmuting the Hamiltonian of the dual model into the initial one. Within our framework, gauging *G* means that we are dealing with MPS tensors of the form of equation (8), where the gauge degrees of freedom depicted by the blue lines are also labelled by irreducible representations of *G*. The building blocks of the MPO for this duality then evaluate to Clebsch–Gordan coefficients. More precisely, for the model in equation (3), where the local Hilbert space is spanned by $\left\vert \underline{3},v\right\rangle$, *v* = 1, …, 3, we have the following identification:15whereby the MPO tensor acts on the space of intertwining maps ${V}_{1}\otimes \underline{3}\to {V}_{2}$. Acting with this MPO then yields an MPS of the form:16We show that, in this very specific case, our procedure amounts to directly simulating the initial phase using symmetry-preserving tensor networks^49–51^. Generally, symmetry-preserving tensor network algorithms exploit a specific expression for the tensors that explicitly enforces the symmetry. Concretely, consider an MPS in the Hilbert space of the model of equation (6). Generically, entanglement degrees of freedom of the MPS tensor live in a vector space *U*. Let us suppose that the MPS tensors are invariant under the action of ${{\mathbb{A}}}_{4}$. As already exploited, the Wigner–Eckart theorem stipulates that the tensors are expressible as linear combination of Clebsch–Gordan coefficients. More concretely, because the vector space *U* is equipped with an ${{\mathbb{A}}}_{4}$ action, it can be decomposed into irreducible representations, that is *U* ≅ ⨁_*V*_〈*U*, *V*〉*V*, where the direct sum is over irreducible representations of ${{\mathbb{A}}}_{4}$ and $\langle V,U\rangle \in {{\mathbb{Z}}}_{\ge 0}$. It follows that we can decompose *u* = 1, …, dim *U* as (*V*, *v*, *d*) ≡ (*V*, *v*) ⊗ (*V*, *d*), where *v* = 1, …, dim *V* and *d* = 1, …, 〈*U*, *V*〉. Using this notation, the MPS tensors decompose as follows:17revealing in particular the sparse block structure of the tensors. As per our graphical calculus, the matrices on the right-hand side labelled by *i* are, in fact, of the form of equation (8). From a symmetric tensor network viewpoint, this decomposition is used to target a specific charge sector of the Hilbert space, thereby reducing computational costs while explicitly enforcing the symmetry^49–51^. Let us now assume that the MPS is the unique ground state of the ${{\mathbb{A}}}_{4}$ symmetric phase. In this case, equation (17) precisely recovers equation (16) under the identification of equation (15) such that the entanglement degrees of freedom of the MPS ground state of the dual model are labelled by pairs (*V*, *d*). One can now verify that our algorithm then produces a result that agrees with symmetry-preserving DMRG. However, on comparing our algorithm to current state-of-the-art implementations, our approach is practically much simpler because it does not require the conventional implementation of the recoupling theory for symmetric tensors based on fusion trees.

As commented in the main text, this decomposition is tailored to the symmetric phase for which entanglement degrees of freedom of the unique ground state transform as linear representations of ${{\mathbb{A}}}_{4}$. By contrast, this is clearly not suited to the ${{\mathbb{A}}}_{4}$ SPT phase, for which entanglement degrees of freedom transform as projective representations of ${{\mathbb{A}}}_{4}$. Indeed, it is well known that using standard symmetric tensor networks in an SPT phase is more costly because it forces the edge modes to transform according to a linear representation, which requires further long-range entanglement^74^.

In practice, most of the utility of symmetry-preserving tensor networks is in models with continuous Lie group symmetries, such as SU(2). Although we restricted ourselves to a finite group, our results readily generalize to these cases, implying, for instance, that a ground state preserving an SU(2) symmetry is most efficiently described in terms of the ground state of a dual model that breaks a dual non-invertible ${\mathsf{Rep}}({\text{SU}}(2))$ symmetry. For this case, the duality transformation recovers the celebrated Schur–Weyl duality. The resulting dual models are the so-called interaction-round-a-face models studied by Sierra and Nishino^61^. Because the different symmetry-broken ground states are related by the action of non-trivial symmetry MPOs, these ground states do not have the same entanglement, which shows the importance of properly initializing the DMRG algorithm to favour the ground states with the least amount of entanglement. Note that the fact that SU(2) admits an infinite number of irreducible representations is practically addressed by assigning a weight zero to higher spin ones, so that we effectively deal with a finite number of blocks only, just as in standard DMRG exploiting symmetry-preserving tensors.

### Mathematical formalism

We summarize here the mathematical formalism underpinning the results presented in the main text. More precise mathematical definitions can be found, for instance, in ref. ^29^. Consider any local one-dimensional quantum lattice model with a generalized symmetry encoded into a fusion category ${\mathcal{C}}$. In the presence of such a generalized symmetry, gapped phases are characterized by a choice of an (indecomposable) ${\mathcal{C}}$-module category, whose objects label the degenerate ground states of the phase^23,36^. The same module categories also characterize the different ways to gauge (sub)symmetries of the model. After performing the gauging operation associated with a ${\mathcal{C}}$-module category ${\mathcal{M}}$, the dual symmetry of the resulting model is encoded into the so-called Morita dual ${{\mathcal{C}}}_{{\mathcal{M}}}^{* }$ of ${\mathcal{C}}$ with respect to ${\mathcal{M}}$. The fusion category ${{\mathcal{C}}}_{{\mathcal{M}}}^{* }$ is defined to be the category ${{\mathsf{Fun}}}_{{\mathcal{C}}}({\mathcal{M}},{\mathcal{M}})$ of ${\mathcal{C}}$-module endofunctors of ${\mathcal{M}}$ (refs. ^29,75^), the fusion structure being provided by the composition of ${\mathcal{C}}$-module functors. Crucially, ${\mathcal{M}}$ is also a ${{\mathcal{C}}}_{{\mathcal{M}}}^{* }$-module category, and we have ${({{\mathcal{C}}}_{{\mathcal{M}}}^{* })}_{{\mathcal{M}}}^{* }\simeq {\mathcal{C}}$. In other words, there is always a way to gauge a subsymmetry of ${{\mathcal{C}}}_{{\mathcal{M}}}^{* }$ so as to recover the initial model.

Let us examine this gauging operation in practice. For conciseness, we focus on nearest-neighbour Hamiltonians, but longer-range interactions can be accommodated just as easily. As for the example studied in the main text, it is crucial to write the Hamiltonian in such a way that the generalized symmetry ${\mathcal{C}}$ is manifest. Under some mild mathematical assumptions about the symmetry MPOs^32,76^, it follows from a generalized Wigner–Eckart theorem^33^ that any local symmetric operator is expressible in terms of generalized Clebsch–Gordan coefficients. Specifically, given a ${\mathcal{C}}$-symmetric Hamiltonian of the form ${\mathbb{H}}=\sum_{{\mathsf{i}} = 1}^{L-1}\sum_{n}{{\mathbb{h}}}_{{\mathsf{i}},n}$, the local operators can always be put in the form18in terms of tensors evaluating to these generalized Clebsch–Gordan coefficients. The graphical notation mimics that of equation (6) and encodes, in particular, that the Hilbert space of a model with a generalized symmetry is generically not a tensor product space. More concretely, there is a (possibly not unique) choice of ${\mathcal{C}}$-module category ${\mathcal{R}}$ such that local operators can be expressed as equation (18), where {*Y*} label objects in ${{\mathcal{C}}}_{{\mathcal{R}}}^{* }$, and the generalized Clebsch–Gordan coefficients are given by the so-called module associator of ${\mathcal{R}}$, as a ${{\mathcal{C}}}_{{\mathcal{R}}}^{* }$-module category (see refs. ^32,34,47^ for details).

It follows from the defining axioms of the ${{\mathcal{C}}}_{{\mathcal{R}}}^{* }$-module category ${\mathcal{R}}$ that the tensors in equation (18) satisfy an analogue of equation (4):19where the *F* symbols ${\left({F}_{{Y}_{4}}^{{Y}_{1}{Y}_{2}{Y}_{3}}\right)}_{{Y}_{5},ij}^{{Y}_{6},kl}\in {\mathbb{C}}$ enter the definition of the fusion category ${{\mathcal{C}}}_{{\mathcal{R}}}^{* }$. Together with the complex coefficients {*h*_*n*_}_*n*_, these *F* symbols provide the structure constants of the algebra generated by the local symmetric operators ${\{{{\mathbb{h}}}_{{\mathsf{i}},n}\}}_{{\mathsf{i}},n}$. Within this framework, performing a gauging operation simply amounts to picking a different ${{\mathcal{C}}}_{{\mathcal{R}}}^{* }$-module category ${{\mathcal{R}}}^{{\prime} }$. This means replacing the tensors in equation (18) by a new set of tensors that now evaluate to generalized Clebsch–Gordan coefficients given by the module associator of ${{\mathcal{R}}}^{{\prime} }$. Crucially, this new set of tensors still satisfy equation (19), so the algebra of local symmetric operators generated by equation (18) is isomorphic to the initial one. The dual symmetry of the resulting model is then encoded into the fusion category ${({{\mathcal{C}}}_{{\mathcal{R}}}^{* })}_{{{\mathcal{R}}}^{{\prime} }}^{* }$. Like the examples discussed in the main text, Hamiltonians associated with different choices of ${{\mathcal{C}}}_{{\mathcal{R}}}^{* }$-module categories can be transmuted into each other through an MPO. Mathematically, such an operator is described by an object in the category ${{\mathsf{Fun}}}_{{{\mathcal{C}}}_{{\mathcal{R}}}^{* }}({\mathcal{R}},{{\mathcal{R}}}^{{\prime} })$ of ${{\mathcal{C}}}_{{\mathcal{R}}}^{* }$-module functors from ${\mathcal{R}}$ to ${{\mathcal{R}}}^{{\prime} }$, in such a way that the building blocks of the MPO are provided by the module structure of such a functor^47^. Moreover, this category of module functors has the structure of a module category ${\mathcal{M}}$ over the symmetry category ${\mathcal{C}}\simeq {{\mathsf{Fun}}}_{{{\mathcal{C}}}_{{\mathcal{R}}}^{* }}({\mathcal{R}},{\mathcal{R}})$ through composition of module functors. We identify this ${\mathcal{C}}$-module category as that encoding the gauging operation corresponding to changing the ${{\mathcal{C}}}_{{\mathcal{R}}}^{* }$-module category ${\mathcal{R}}$ into ${{\mathcal{R}}}^{{\prime} }$ in such a way that the dual symmetry is encoded into ${{\mathcal{C}}}_{{\mathcal{M}}}^{* }\simeq {({{\mathcal{C}}}_{{\mathcal{R}}}^{* })}_{{{\mathcal{R}}}^{{\prime} }}^{* }$. Note that for many fusion categories of interest, indecomposable module categories have been classified. Importantly, the data of the module functors needed to describe the MPO intertwiners relating the different dual models can be obtained as a representation theoretic problem^33,77^, which numerically can be reduced to a linear algebra problem.

Let us now suppose that the initial ${\mathcal{C}}$-symmetric model, which is defined with respect to the ${{\mathcal{C}}}_{{\mathcal{R}}}^{* }$-module category ${\mathcal{R}}$, is in the phase associated with the ${\mathcal{C}}$-module category ${\mathcal{P}}$. Our goal is to find a dual model whose dual symmetry is completely broken in the ground-state subspace. To achieve this, we must understand how to relate the phase of the dual model to the phase of the initial model, which is not immediate given that the symmetry structures differ. To this end, we can think of the ${\mathcal{C}}$-module category ${\mathcal{P}}$ as ${{\mathsf{Fun}}}_{{{\mathcal{C}}}_{{\mathcal{R}}}^{* }}({\mathcal{R}},{\mathcal{Q}})$ for some ${{\mathcal{C}}}_{{\mathcal{R}}}^{* }$-module category ${\mathcal{Q}}$. Indeed, because ${\mathcal{C}}\simeq {{\mathsf{Fun}}}_{{{\mathcal{C}}}_{{\mathcal{R}}}^{* }}({\mathcal{R}},{\mathcal{R}})$, it does define a ${\mathcal{C}}$-module category through composition of module functors. Because any ${\mathcal{C}}$-module category can be constructed in this way^29^, it follows that ${\mathcal{P}}$ uniquely fixes ${\mathcal{Q}}$. The action of a duality operator ${{\mathsf{Fun}}}_{{{\mathcal{C}}}_{{\mathcal{R}}}^{* }}({\mathcal{R}},{{\mathcal{R}}}^{{\prime} })$ on the phase ${\mathcal{P}}$ can now be obtained through composition of module functors, such that the new phase is encoded into ${{\mathsf{Fun}}}_{{{\mathcal{C}}}_{{\mathcal{R}}}^{* }}({{\mathcal{R}}}^{{\prime} },{\mathcal{Q}})$, which is indeed a module category over the dual symmetry ${({{\mathcal{C}}}_{{\mathcal{R}}}^{* })}_{{{\mathcal{R}}}^{{\prime} }}^{* }={{\mathsf{Fun}}}_{{{\mathcal{C}}}_{{\mathcal{R}}}^{* }}({{\mathcal{R}}}^{{\prime} },{{\mathcal{R}}}^{{\prime} })$. The optimal dual model, whose dual symmetry is completely broken, is obtained when the module category that describes the dual phase is given by the dual symmetry itself. From the above, it is clear that this amounts to choosing ${{\mathcal{R}}}^{{\prime} }={\mathcal{Q}}$ so that ${\mathcal{Q}}$ encodes the degrees of freedom. Consider a variational ground-state MPS in the constrained Hilbert space of the optimal dual model with degrees of freedom in ${\mathcal{Q}}$, which maximally breaks the dual symmetry. The remaining symmetry-breaking ground states can be obtained by acting with symmetry MPOs labelled by simple objects in ${{\mathcal{C}}}_{{\mathcal{P}}}^{* }$ of the form20where the individual tensors are determined by the data of the relevant ${{\mathcal{C}}}_{{\mathcal{R}}}^{* }$-module functors^47^. Finally, these dual ground states can be mapped to ground states of the original model, which is defined with respect to the ${{\mathcal{C}}}_{{\mathcal{R}}}^{* }$-module category ${\mathcal{R}}$, by acting with a duality MPO:21Conversely, the duality operators in the optimal model are encoded into ${{\mathsf{Fun}}}_{{{\mathcal{C}}}_{{\mathcal{R}}}^{* }}({\mathcal{R}},{\mathcal{Q}})$, which is equivalent to the ${\mathcal{C}}$-module category ${\mathcal{P}}$ encoding the phase of the original model. The various fusion categories and the Morita equivalences between them, both before and after the duality, can be summarized as22

To conclude, we revisit our example in light of this general formalism. When dealing with an ordinary (invertible) symmetry ${{\mathbb{A}}}_{4}$, the corresponding fusion category ${\mathcal{C}}$ is the category ${{\mathsf{Vec}}}_{{{\mathbb{A}}}_{4}}$ of ${{\mathbb{A}}}_{4}$-graded vector spaces. The different ways to gauge subsymmetries of ${{\mathbb{A}}}_{4}$ are provided by ${{\mathsf{Vec}}}_{{{\mathbb{A}}}_{4}}$-module categories, which are known to be classified by pairs (*A*, [*ψ*]), as defined in the main text^69^. In particular, choosing *A* = *G* and *ψ* = 1 amounts to the (untwisted) gauging of *A*, and the corresponding ${{\mathsf{Vec}}}_{{{\mathbb{A}}}_{4}}$-module category is equivalent to the category ${\mathsf{Vec}}$ of complex vector spaces. The Morita dual ${({{\mathsf{Vec}}}_{{{\mathbb{A}}}_{4}})}_{{\mathsf{Vec}}}^{* }$ of ${{\mathsf{Vec}}}_{{{\mathbb{A}}}_{4}}$ with respect to ${\mathsf{Vec}}$ can be confirmed to be equivalent to the category ${\mathsf{Rep}}({{\mathbb{A}}}_{4})$ of representations of ${{\mathbb{A}}}_{4}$, in agreement with our results. When writing the Hamiltonian as in equation (1), we are choosing the ${\mathsf{Rep}}({{\mathbb{A}}}_{4})$-module category ${\mathcal{R}}={\mathsf{Vec}}$ such that the module associator boils down to the ordinary Clebsch–Gordan coefficients of ${{\mathbb{A}}}_{4}$. We obtain the various dual models by choosing different ${\mathsf{Rep}}({{\mathbb{A}}}_{4})$-module categories. Specifically, the dual model resulting from the *ψ*-twisted gauging of *H* is obtained by choosing the module category ${{\mathcal{R}}}^{{\prime} }={{\mathsf{Rep}}}^{\psi }(H)$ of *ψ*-projective representations of *H*, and the dual symmetry is encoded into ${({\mathsf{Rep}}({{\mathbb{A}}}_{4}))}_{{{\mathsf{Rep}}}^{\psi }(H)}^{* }$.

We now suppose that the initial model is in the phase associated with the ${{\mathsf{Vec}}}_{{{\mathbb{A}}}_{4}}$-module category ${\mathcal{M}}(K,\phi ):={{\mathsf{Fun}}}_{{\mathsf{Rep}}({{\mathbb{A}}}_{4})}({\mathsf{Vec}},{{\mathsf{Rep}}}^{\phi }(K))$. The ${{\mathbb{A}}}_{4}$ SPT, ${{\mathbb{A}}}_{4}$ symmetric and ${{\mathbb{D}}}_{2}$ symmetric phases considered in the main text were obtained by choosing ${{\mathsf{Rep}}}^{\phi }(K)$ to be equal to ${{\mathsf{Rep}}}^{\psi }({{\mathbb{A}}}_{4})$, ${\mathsf{Rep}}({{\mathbb{A}}}_{4})$ and ${\mathsf{Rep}}({{\mathbb{D}}}_{2})$, respectively. The optimal model is always found to be that given by ${{\mathcal{R}}}^{{\prime} }={{\mathsf{Rep}}}^{\phi }(K)$, which amounts to performing a *ϕ*-twisted gauging of *K*, as predicted. The relevant Morita equivalences can be summarized as23where the phase is encoded in the ${{\mathsf{Vec}}}_{{{\mathbb{A}}}_{4}}$-module category ${\mathcal{M}}(K,\phi )$, which states that the different ground states are labelled by cosets in *G*/*K* and the remaining symmetry *K* acts *ϕ*-projectively.

Let us also shed light on some of the suboptimal simulations. For instance, consider the ${{\mathbb{A}}}_{4}$-symmetric phase and the dual model labelled by ${{\mathcal{R}}}^{{\prime} }={{\mathsf{Rep}}}^{\psi }({{\mathbb{D}}}_{2})$. The symmetry is ${({\mathsf{Rep}}({{\mathbb{A}}}_{4}))}_{{{\mathsf{Rep}}}^{\psi }({{\mathbb{D}}}_{2})}^{* }\simeq {{\mathsf{Vec}}}_{{{\mathbb{A}}}_{4}}$, whereas the dual phase is that associated with the ${{\mathsf{Vec}}}_{{{\mathbb{A}}}_{4}}$-module category ${{\mathsf{Fun}}}_{{\mathsf{Rep}}({{\mathbb{A}}}_{4})}({{\mathsf{Rep}}}^{\psi }({{\mathbb{D}}}_{2}),{\mathsf{Rep}}({{\mathbb{A}}}_{4}))\simeq {\mathsf{Vec}}$. Moreover, because ${{\mathsf{Rep}}}^{\psi }({{\mathbb{D}}}_{2})$ is equivalent to ${\mathsf{Vec}}$ as a category, this explains why the numerical results were the same for this dual model as for the initial one. In a similar vein, in the ${{\mathbb{A}}}_{4}$ SPT phase, the dual model obtained by choosing ${{\mathcal{R}}}^{{\prime} }={\mathsf{Rep}}({{\mathbb{D}}}_{2})$ has a ${({\mathsf{Rep}}({{\mathbb{A}}}_{4}))}_{{\mathsf{Rep}}({{\mathbb{D}}}_{2})}^{* }\simeq {{\mathsf{Vec}}}_{{{\mathbb{A}}}_{4}}$ symmetry. The dual phase is associated with the ${{\mathsf{Vec}}}_{{{\mathbb{A}}}_{4}}$-module category ${{\mathsf{Fun}}}_{{\mathsf{Rep}}({{\mathbb{A}}}_{4})}({\mathsf{Rep}}({{\mathbb{D}}}_{2}),{{\mathsf{Rep}}}^{\psi }({{\mathbb{A}}}_{4}))$, which is equivalent to ${\mathsf{Vec}}$ as a category, meaning that the whole symmetry is preserved, in agreement with our numerical results. Finally, let us examine the ${{\mathbb{D}}}_{2}$ symmetric phase and the dual model obtained by choosing the ${{\mathsf{Vec}}}_{{{\mathbb{A}}}_{4}}$-module category ${{\mathcal{R}}}^{{\prime} }={\mathsf{Rep}}({{\mathbb{Z}}}_{3})$. The dual symmetry ${({\mathsf{Rep}}({{\mathbb{A}}}_{4}))}_{{\mathsf{Rep}}({{\mathbb{Z}}}_{3})}^{* }\simeq {\mathsf{Rep}}({{\mathbb{A}}}_{4})$ is also fully preserved because the module category over it is ${{\mathsf{Fun}}}_{{\mathsf{Rep}}({{\mathbb{A}}}_{4})}({\mathsf{Rep}}({{\mathbb{Z}}}_{3}),{\mathsf{Rep}}({{\mathbb{D}}}_{2}))$, which happens to be equivalent to ${\mathsf{Vec}}$, in agreement with our numerical results.

## Online content

Any methods, additional references, Nature Portfolio reporting summaries, source data, extended data, supplementary information, acknowledgements, peer review information; details of author contributions and competing interests; and statements of data and code availability are available at 10.1038/s41567-025-02961-2.

## Source data

Source Data Fig. 1 Raw entanglement spectra and memory requirements.

Source Data Fig. 2 Raw entanglement spectra and memory requirements.

Source Data Fig. 3 Raw entanglement spectra and memory requirements.

## Data Availability

All relevant data are included in the plots and are available at https://github.com/lalooten/DualityDMRG.

## Code availability

MATLAB code for computing the ground states of the various models and their corresponding entanglement spectra is available at https://github.com/lalooten/DualityDMRG.
